# Association between serum heavy metal levels and diabetic retinopathy in NHANES 2011–2020

**DOI:** 10.1038/s41598-024-51749-6

**Published:** 2024-01-13

**Authors:** Yan Zhang, Xuekui Liu, Xia Zhang, Lin Li, Qing Li, Houfa Geng, Li Shi, Ben Wang, Qinqin Qiu, Tianpei Yu, Yiquan Sang, Liying Wang, Jun Liang, Wei Xu

**Affiliations:** 1 The Xuzhou Clinical College of Xuzhou Medical University, Xuzhou, Jiangsu China; 2 Department of Endocrinology, Xuzhou Central Hospital, Xuzhou Institute of Medical Sciences, Xuzhou Clinical School of Nanjing Medical University, Affiliated Hospital of Medical School of Southeast University, Xuzhou, Jiangsu China; 3 Bengbu Medical College, Bengbu, Anhui China

**Keywords:** Environmental sciences, Environmental social sciences, Endocrinology, Medical research, Risk factors

## Abstract

The present study utilized the National Health and Nutrition Examination Survey (NHANES) database to examine the relationship between serum levels of heavy metals and Diabetic retinopathy (DR) in individuals aged over 30 years with type 2 diabetes mellitus (T2DM) in the United States. A cross-sectional analysis was conducted on 1583 individuals with T2DM from the NHANES 2011–2020, including 331 individuals in the DR group and 1252 individuals in the non-DR group. We collected data on serum levels of heavy metals, DR, and serum albumin for descriptive statistics, linear regression, and logistical regression analysis. After adjusting for age, gender, race and other factors, there was no statistically significant association between blood cadmium, selenium, mercury, or lead and DR. However, serum manganese (Mn) and DR had a significant negative association (β = − 0.2045, 95% CI = − 0.3484, − 0.0606). Serum albumin partially modulated the indirect influence of serum Mn on the incidence of DR, accounting for 12.80% of the association between serum Mn and DR. There was a negative association between serum Mn levels and the prevalence of DR in people with T2DM. Mn intake at least in this study has a little influence on the onset and development of DR.

## Introduction

Diabetes mellitus (DM) is a major public health problem worldwide and one of the major diseases threatening human health and survival. Diabetic retinopathy (DR) is the most common microvascular complication of diabetes and one of the leading causes of blindness in people with diabetes in clinical practice, affecting people’s quality of life^1–3^. The biochemical mechanisms underpinning the development of DR are poorly understood and may be related to metabolic abnormalities, cellular signaling pathway transduction, glucotoxicity, and oxidative stress^4^. Although accumulating epidemiological evidence has linked serum metal exposure to diabetes, the association between heavy metals and DR remains elusive.

Due to the rapid development of industrialization and urbanization, the pollution concentration of lead (Pb), cadmium (Cd), mercury (Hg), selenium (Se), and manganese (Mn) in the environment is higher than that of other heavy metals^5,6^. Epidemiologic studies that have evaluated the relationship between these heavy metals and the risk of type 2 diabetes mellitus (T2DM) and its complications. An observational study showed that higher Mn intake was directly associated with a lower risk of type 2 diabetes in a population of postmenopausal women^7^. Previous study found a negative association between typical levels of serum Cd and diabetes risk in a United States of America population aged 20 years or older^8^. Kornhauser et al. reported that plasma Se was negatively associated with the severity of diabetic nephropathy in patients with type 2 diabetes^9^. There exists scientific evidence indicating that low levels of Cd exposure exacerbate the progression of kidney disease in diabetics^10^. However, the relationship between these heavy metals and diabetic retinopathy is poorly studied. Serum albumin (ALB) is a crucial physiological transporter of vital metal ions Pb^2+^, Cd^2+^, Hg^2+^, Se^2+^, and Mn^2+^ in the bloodstream^11,12^. Nonetheless, the evidence as to whether heavy metals influence the incidence of DR by binding to ALB and thereby affecting the incidence of DR evidence on the relationship between serum ALB and heavy metals and DR is unclear.

Therefore, we hypothesized that the relationship between blood levels of these five heavy metals and DR is nonlinear. The present sought to explore the association between blood levels of Pb, Cd, Hg, Se, Mn, and DR using data from a representative sample of people with T2DM who participated in the National Health and Nutrition Examination Survey (NHANES) between 2011 and 2020.

## Methods

### Sample and overall

The National Center for Health Statistics (NCHS) conducted a population-based cross-sectional NHANES to collect data on the nutritional and health conditions of children and adults in the United States of America via interviews, physical examinations, and laboratory tests. NHANES employs a categorized, multistage probability sampling design to select a sample of an unstructured population to evaluate health and nutritional status. NHANES procedures and protocols were approved by the NCHS Research Ethics Review Committee and written consent was obtained.

### Inclusion and exclusion criteria

**Figure 1:**
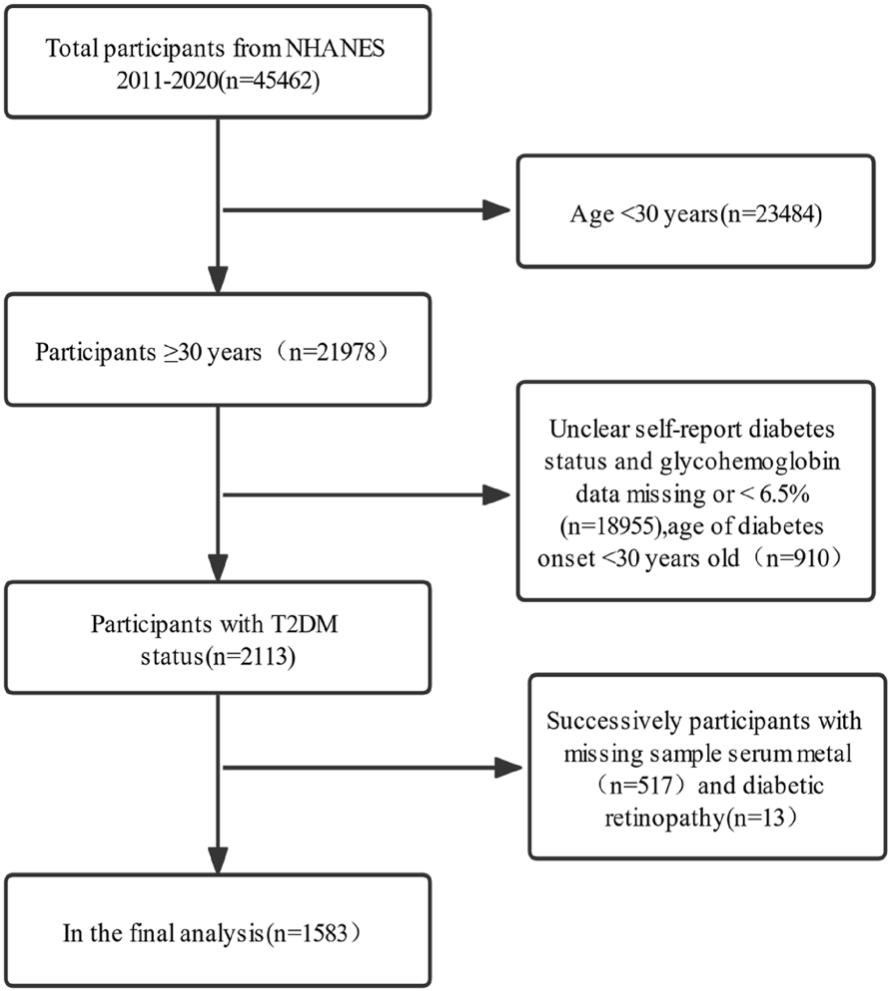
Flowchart of study target population NHANES (2011–2020).

The study population was mainly over 30 years of age. This cross-sectional analysis used data from five NHANES survey cycles (each lasting two years) between 2011 and 2020. Of the 45,462 participants in this study, participants aged < 30 years (n = 23,484), those with unknown diabetes-related conditions, and those lacking information on the glycated hemoglobin (HbA1c) state or the HbA1c level was less than 6.5% (n = 18,955) were eliminated. Diabetic patients aged < 30 years (n = 910) were eliminated to lower the likelihood of having type 1 diabetes. Besides, participants lacking information on serum levels of heavy metals (n = 517) and DR (n = 13) were disqualified. We filled in the remaining missing values in using the average of the entire sequence in the spss software. Finally, 1583 participants were included in the analysis. The study design flow is depicted in Fig. 1.

### Diagnosis of DR

The following criteria were used to identify diabetes: (1) a prior diagnosis from a medical expert, (2) fasting blood glucose (FBG) levels of > 7.0 mmol/L, (3) HbA1c levels of > 6.5%, or (4) receiving diabetes medication^13^. DR was self-reported using a dichotomous classification, whereby the respondent had been informed by their physician that diabetes was affecting their eyes.

### Determination of heavy metals

Whole blood specimens were collected by physicians at the NHANES Mobile Examination Center (MEC) via venipuncture in EDTA-coated tubes, centrifuged on-site, and stored frozen at − 30 °C for transportation to the Centers for Disease Control and Prevention (CDC) laboratory in California, where they were stored in a frozen state until further analysis. First, in the sample dilution step, a small amount of whole blood is taken from a larger sample and mixed to distribute the cellular components evenly. This mixing is critical because certain metals (e.g., lead) are primarily associated with red blood cells. Samples with clots or microclots are identified and excluded from analysis due to concerns about sample inhomogeneity. Diluted blood samples are prepared by mixing 1 part of the sample, 1 part of water, and 48 parts of diluent. The diluent contains chemicals that release metals from red blood cells, reduce ionization inhibition, prevent clogging, and enable the use of internal standards. The diluted sample is then passed through an inductively coupled plasma (ICP) ion source into the mass spectrometer. The liquid blood sample is converted into aerosol droplets that are vaporized, atomized, and ionized in the plasma region. The resulting ions enter the mass spectrometer with argon gas for analysis. The Dynamic Reaction Cell (DRC) plays a vital role in the selective reaction by removing interferences or enhancing the ion signal of specific elements. The ions passing through the DRC are electrically selected and directed to the analytical quadrupole. The electrical signal generated by the ion impact detector is processed into digital information to determine the elemental concentration. For the values under LODs, an imputed fill value (LOD/√2) was adopted to fill up the vacancy in data preprocessing. Quality assessment and quality control (QA/QC) processes for data collection from blood samples complied with the Therapeutic Laboratory Development Act of 1988^14^. Detail information of the NHANES laboratory procedure is available at https://www.cdc.gov/nchs/nhanes/index.htm.

### Covariates

Data on demographics (age, gender, and race/ethnicity), academic achievement, poverty-to-income proportion, smoking, body mass index (BMI), waist circumference, serum ALB, FBG, HbA1c, triglycerides, and total cholesterol were collected using standardized questionnaires. Participants were divided into Caucasian, Black, Hispanic, Mexican American, and other race categories. Height (H, m) and weight (W, kg) were measured according to norms, and BMI was calculated as W/H^2^ (kg/m^2^). Smokers were defined as individuals who smoked ≥ 100 cigarettes^15^. Smoking status was divided into three categories: never smoked (< 100 cigarettes in their lifetime), gave up smoking (previously smoked > 100 cigarettes but no longer smoke), and still smoking (previously smoked > 100 cigarettes and still smoking). Hypertension was defined as systolic blood pressure > 140 mmHg or diastolic blood pressure > 90 mmHg, use of blood pressure drugs, or self-reported high blood pressure^16^.

### Statistical analysis

Categorical variables were expressed as proportions and frequencies and were statistically analyzed using Chi-square analyses. Continuous variables were expressed as mean and standard errors and were analyzed using linear regression models. Multifactorial logistic regression analysis was used to examine the relationship between heavy metal exposure and DR. Additionally, subgroup analysis of heavy metal concentration and DR was performed to test whether the effect of serum levels of heavy metal on DR could be altered by age, sex, and BMI. All data analyses were performed using R (version 4.0.2) and SPSS software (version 24.0). p < 0.05 was considered statistically significant.

### Institutional review board statement

Our study used five cycles of open NHANES database (2011–2020), National Center for Health Statistics granted the study procedures of Ethics Review Board. These data could be accessible at the following URL: https://www.cdc.gov/nchs/nhanes/irba98.htm#print.

### Ethics statement

All survey participants signed a declaration of consent form after being made aware of the nature of the poll. The informed consent was approved after evaluation by the Committee of the National Center for Statistics on Health Ethics Assessment Board. To make the best use of these resources, all of the data is made publicly accessible after formal anonymization. These data could be accessible at the following URL: https://www.cdc.gov/nchs/nhanes/irba98.htm#print.

## Results

### Participant characteristics

This study included 1583 T2DM patients, of which 331 had concomitant DR and 1252 had no concomitant DR. The demographic details of participants with or without DR are shown in Table 1. DR participants were older and had higher HbA1c levels and lower waist circumference, BMI, serum ALB, and serum Mn levels than non-DR participants. No statistically significant difference was observed between DR and non-DR groups in terms of race, gender, education, household income-to-poverty ratio, triglycerides, total cholesterol, smoking status, blood Pb, Cd, Hg, and Se, and hypertension status (p > 0.05).

**Table 1:** Characteristics of study sample with and without diabetic retinopathy.

	Non-diabetic retinopathy (n = 1252)	Diabetic retinopathy (n = 331)	p-value
Age, years	62.85 ± 11.05	64.5 ± 10.68	0.015
Sex, n (%)			0.700
Male	707 (56.5%)	183 (55.3%)	
Female	545 (43.5%)	148 (44.7%)	
Race, n (%)			0.281
Mexican American	195 (15.6%)	48 (14.5%)	
Other Hispanic	124 (9.9%)	44 (13.3%)	
White	393 (31.4%)	89 (26.9%)	
Black	351 (28.0%)	97 (29.3%)	
Other race	189 (15.1%)	53 (16.0%)	
Educational level, n (%)			0.463
Less than high school	372(29.7%)	109(32.9%)	
High school	296(23.6%)	82(24.8%)	
More than high school	582(46.5%)	140(42.3%)	
Don’t know	2(0.2%)	0(0.0%)	
Ratio of family income to poverty	2.36 ± 1.56	2.16 ± 1.51	0.054
Smoking status			0.263
Current smoker	194 (15.5%)	40 (12.1%)	
Never smoker	630 (50.3%)	178 (53.8%)	
Former smoker	428 (34.2%)	113 (34.1%)	
Hypertension			0.384
Yes	860(68.7%)	235 (71.0%)	
No	389(31.1%)	94 (28.4%)	
Don’t know	3 (0.2%)	2 (0.6%)	
Waist circumference (cm)	110.17 ± 16.00	107.56 ± 14.75	0.011
BMI (kg/m^2^)	32.38 ± 7.28	31.23 ± 6.47	0.007
Serum glucose (mg/dL)	167.84 ± 75.86	177.61 ± 84.42	0.036
Glycohemoglobin (%)	8.07 ± 1.68	8.33 ± 1.65	0.013
Triglyceride (mg/dL)	1.81 ± 1.61	1.60 ± 1.27	0.119
Total-cholesterol (mg/dL)	4.63 ± 1.20	4.59 ± 1.32	0.546
Serum albumin (g/dL)	4.08 ± 0.35	4.00 ± 0.41	< 0.001
Blood lead (μmol/L)	0.06 ± 0.06	0.06 ± 0.05	0.416
Blood cadmium (nmol/L)	4.16 ± 4.90	4.00 ± 4.34	0.586
Blood mercury, total (μmol/L)	7.44 ± 14.79	7.16 ± 14.21	0.758
Blood selenium (μmol/L)	2.46 ± 0.40	2.42 ± 0.35	0.125
Blood manganese (μmol/L)	173.20 ± 69.02	162.99 ± 62.80	0.015

Continuous variables are presented as mean ± SD, p-value was calculated by a linear regression model. Categorical variables are presented as %, p-value was calculated by Chi-square test.

### Relationship between serum level of heavy metal and DR

Multifactorial logistic regression analysis was performed to evaluate the relationship between serum heavy metal exposure and DR, as shown in Table 2. The data from Model 1 was left unadjusted, Model 2 was adjusted for gender, age, and race, and Model 3 was adjusted for education, household income-to-poverty ratio, smoking, hypertension, and BMI based on Model 2. The blood Mn exposure in Model 1 decreased the incidence of DR (p = 0.002). The blood Mn exposure was significantly negatively associated with DR in Models 2 and 3. However, no association was observed between blood Cd, Se, Hg, or Pb exposure and DR in all models (p > 0.05).

**Table 2:** Association between blood metal levels and DR.

	Model 1, β (95% CI)	p	Model 2, β (95% CI)	p	Model 3, β (95% CI)	p
DR						
Blood lead (μmol/L)	0.0062 (− 0.0665, 0.0788)	0.867	− 0.0160 (− 0.0923, 0.0602)	0.680	− 0.0307 (− 0.1179, 0.0565)	0.490
Blood cadmium (nmol/L)	0.0024 (− 0.0554, 0.0602)	0.934	− 0.0067 (− 0.0657, 0.0523)	0.825	− 0.0201 (− 0.0892, 0.0490)	0.569
Blood mercury, total (μmol/L)	− 0.0203 (− 0.0652, 0.0246)	0.375	− 0.0216 (− 0.0682, 0.0249)	0.362	− 0.0168 (− 0.0697, 0.0362)	0.535
Blood selenium (μmol/L)	− 0.2291 (− 0.5376, 0.0794)	0.145	− 0.189962 (− 0.5005, 0.1206)	0.230	− 0.1766 (− 0.5240, 0.1709)	0.319
Blood manganese (μmol/L)	− 0.2016 (− 0.3309, − 0.0724)	0.002	− 0.19230 (− 0.3230, − 0.0629)	0.004	− 0.2045 (− 0.3484, − 0.0606)	0.005

Model 1: no modification variables; Model 2: only adjusts for age, gender, and race; Model 3: adjusts age, gender, race, educational level, ratio of family income to poverty, skiing status, hypertension, BMI, and waist circumference.

Logarithmic conversion of all blood metal levels data.

### Subgroup analysis

A significant negative correlation between blood Mn content and DR was observed after adjusting for age, sex, ethnicity, and other factors. The subgroups were further stratified by age, sex, and BMI, as shown in Table 3. In the age ≥ 60 years group, blood Mn was significantly negatively associated with DR prevalence without adjusting for confounders (p < 0.001) and even after adjusting for all potential variables. However, no significant association between blood Mn and DR in the 30–44 and 45–60 age categories (p > 0.05). After adjusting for all variables, the data were stratified by gender, and the results showed there was a significant negative association between blood Mn concentration and DR prevalence in the male population compared with the female population (p = 0.013). When stratified by BMI, no significant correlations were observed between blood Mn and DR in 0–25 and 25–30 kg/m^2^ groups after adjusting for confounders. Additionally, a significant inverse correlation was found between blood Mn and DR in the BMI ≥ 30 kg/m^2^ group (p = 0.014).

**Table 3:** Association between serum manganese and stratified by age, sex, and BMI.

	Model 1, β (95% CI)	p-value	Model 2, β (95% CI)	p-value	Model 3, β (95% CI)	p-value
DR						
Stratified by age						
30–44 years	0.1198 (− 0.3538, 0.5935)	0.617	0.2014 (− 0.3115, 0.714)	0.438	0.3312 (− 0.1980, 0.8603)	0.216
45–59 years	0.0010 (− 0.2336, 0.2536)	0.936	0.0206 (− 0.2256, 0.2668)	0.869	− 0.0180 (− 0.3003, 0.2644)	0.901
≥ 60 years	− 0.3092 (− 0.4704, − 0.1480)	< 0.001	− 0.3119 (− 0.4735, − 0.1503)	< 0.001	− 0.3122 (− 0.4903, − 0.1342)	0.001
Stratified by sex						
Male	− 0.2215 (− 0.3960, − 0.0469)	0.013	− 0.2203 (− 0.3949, − 0.0456)	0.013	− 0.2533 (− 0.4526, − 0.0540)	0.013
Female	− 0.1857 (− 0.3801, 0.0087)	0.061	− 0.1615 (− 0.3574, 0.0345)	0.106	− 0.1418 (− 0.3501, 0.0665)	0.182
Stratified by BMI (kg/m^2^) (%)						
0–25	− 0.0544 (− 0.3948, 0.2860)	0.753	− 0.1397 (− 0.5133, 0.2339)	0.462	− 0.1570 (− 0.5634, 0.2494)	0.447
25–30	− 0.1441 (− 0.3954, 0.1072)	0.261	− 0.1303 (− 0.3827, 0.1221)	0.311	− 0.1667 (− 0.4411, 0.1078)	0.233
≥ 30	− 0.2633 (− 0.4333, − 0.0934)	0.002	− 0.2553 (− 0.4289, − 0.0817)	0.004	− 0.2422 (− 0.4351, − 0.0493)	0.014

Model 1: no modification variables; Model 2: only adjusts for age, gender, and race; Model 3: adjusts age, gender, race, educational level, ratio of family income to poverty, smoking status, hypertension, BMI, and waist circumference.

The model is not adjusted for the stratification variable itself in the subgroup analysis.

Logarithmic conversion of all serum manganese data.

### Effect of mediating factors on the association between serum Mn and DR

**Figure 2:**
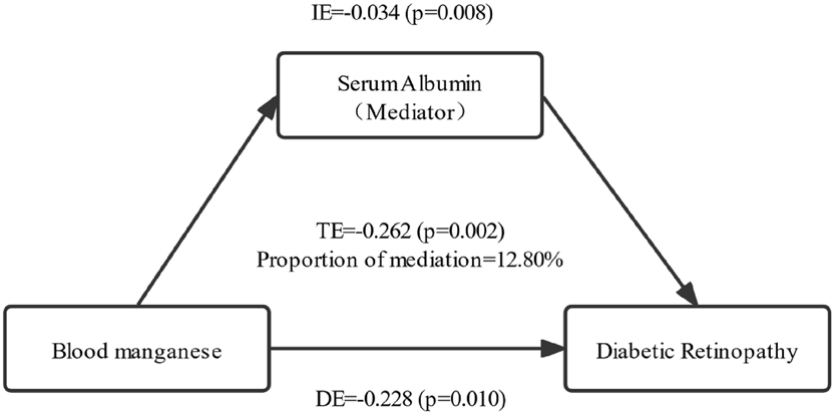
Effect of the serum albumin (mediator) on the relationship between blood manganese (exposure) and diabetic retinopathy (DR outcome); *DE* direct effect, *IE* indirect effect, *TE* total effect.

The Baron and Kenny causal step method combined with multiple linear regression models was utilized to analyze the mediating role of serum ALB in the association between serum Mn and DR. As shown in Fig. 2, after adjusting for Model 3, serum Mn had a direct impact on the prevalence of DR (p = 0.010), which was partially mediated by ALB (p = 0.008). Serum ALB mediated 12.80% of the association between blood Mn and DR.

## Discussion

The present study aimed to elucidate whether there was an association between serum levels of heavy metals and DR among individuals with T2DM in the United States of America. We found a substantial negative relationship between blood Mn and DR, which was partially mediated by serum ALB. Only individuals aged 60 years exhibited a significant inverse association between blood Mn levels and DR after age stratification. Under sex stratification, a significant negative association between blood Mn levels and DR prevalence was observed in the male population compared with the female population. Moreover, a statistically significant association between blood Mn and DR was observed in the BMI ≥ 30 kg/m^2^ group under BMI stratification.

DR, a common microvascular complication of diabetes with a global prevalence of 34.6%, is a major cause of blindness^17^. The onset and evolution of DR are influenced by various mechanisms, such as inflammatory processes, high blood pressure, abnormal lipid metabolism, and insulin resistance^18–20^. Only a few studies have examined the association between exposure to heavy metals and DR, and the findings are controversial. Pb, Hg, Se, and Cd are widespread environmental heavy metal toxicants. Handan et al. found that Se exerts a protective effect on retinal pigment epithelium (ARPE-19) and primary human retinal microvascular endothelial (ACBRI 181) cells against high glucose (HG)-induced oxidative stress and apoptosis^21^. A previous retrospective study found that blood Cd levels were higher in the DR group than in the DM group^22^. These findings are inconsistent with our results, perhaps due to different populations, races, disease states, and health conditions that affect the absorption, transport, and metabolism of Se and Cd in the body, affecting the association between Se and Cd and the disease.

Mn is one of the essential components of the Mn super antioxidant dismutase (MnSOD), which scavenges reactive oxygen species (ROS) under mitochondrial oxidative stress. MnSOD genes and Mn levels may impact MnSOD activity^23^. It was previously demonstrated that Mn administration improved MnSOD activation and shielded people from T2DM and related consequences^24^. Kowluru et al. demonstrated that Mn-SOD overexpression prevented glucose-induced increased oxidative stress and apoptosis of retinal endothelial cells, suggesting a protective role for Mn-SOD in the pathogenesis of diabetic retinopathy^25^. On the other hand, previous studies have found that insulin synthesis and secretion in the pancreas are impaired, and insulin degradation and glucagon release are accelerated in Mn-deficient mice, whereas other studies have found that Mn reduces oxidative stress through antioxidant enzyme systems and non-enzymatic pathways to protect pancreatic islet cells from reactive oxygen species (ROS)^26,27^. The current study suggested that blood Mn may act as a barrier against the onset of DR. This is consistent with our findings. However, whether Mn can influence the development of DR through other metabolic pathways has yet to be investigated.

In plasma, approximately 80% of the oxidized state of Mn^2+^ is bound to ALB and globulin and transported to the liver, kidney, small intestine, endocrine glands, pancreas, brain, bone, muscle, and hair^12,28^. ALB is a glycosylated protein synthesized and secreted by hepatocytes, which has anti-oxidative stress and anti-inflammatory^29^. Previous studies have shown reduced serum ALB levels are associated with DR risk^30,31^. The present study found that serum ALB significantly mediated the association between blood Mn and DR. This implies that blood manganese combined with serum albumin may reduce the development of DR through its antioxidant and anti-inflammatory effects.

In this study, we found a negative correlation between blood Mn and DR only in people aged ≥ 60 years. This may be because aging is linked to a decline in Mn levels. This generally agrees with a large cross-sectional study^32^, which found that serum Mn levels in people aged ≥ 60 years may contribute to preventing and controlling prediabetes and diabetes. Compared with females, males in the current study exhibited an adverse correlation between serum Mn levels and DR. According to a survey of dietary intake of Mn, females received considerably more Mn from their diets than males^33^. The survey also showed that the biological half-life of Mn was substantially lower in women than in men. These results imply that Mn absorption and metabolism differ between males and females, which may help to explain some of the observed gender disparities in our study.

Our study is the first study to explore the relationship between blood levels of heavy metals and DR in T2DM patients older than 30 in the United States of America. Nonetheless, the current study has some limitations. First, we could not demonstrate the causal link between specific molecular mechanisms in blood Mn and DR due to the cross-sectional design of the study, which should be further validated in a prospective cohort study and fundamental research. Second, the outcome variable was based on self-reported history of DR, which may not be entirely accurate. Third, although some of the confounders were adjusted in this study, there were still many confounders affecting DR, such as drug use and family history of dyslipidemia, that we were unable to obtain. Finally, because the NHANES-based study population was used, it was difficult to assess the validity of the findings from various ethnic studies.

## Conclusions

In summary, the present study found a significant negative association between blood Mn levels and DR, suggesting that an appropriate increase in Mn intake may delay the onset and development of DR.

## Data Availability

The data used in the present research were obtained from publicly accessible sources. These data could be accessible at the following URL: https://www.cdc.gov/nchs/nhanes/.
